# Oral Health and Nutrition in Older Adults

**DOI:** 10.3390/nu17193046

**Published:** 2025-09-24

**Authors:** Florence Mei Fung Wong, Wai Keung Leung

**Affiliations:** 1 School of Nursing, Tung Wah College, Hong Kong SAR, China; florencewong@twc.edu.hk; 2 Faculty of Dentistry, The University of Hong Kong, Hong Kong SAR, China

We are pleased to present this Special Issue, “Oral Health and Nutrition in Older Adults,” which features nine insightful publications that reflect the current landscape of clinical practice and scientific evidence. These contributions deepen our understanding of the intricate relationship between oral health, nutrition, and overall well-being among the aging population—a crucial area of study as global demographics shift toward an increasingly older society.

The majority of these studies are original research articles from diverse countries, illustrating the universal nature of oral health challenges faced by older adults worldwide. Regardless of differing healthcare systems and cultural contexts, issues such as dental caries, periodontal disease, edentulism, and impaired oral function are widespread. This geographic diversity underscores that poor oral health is a global concern, affecting the quality of life, nutritional intake, and overall health across populations.

Complementing these original studies, a comprehensive review synthesizes recent advancements in oral treatments, with a particular focus on functional neuroplasticity with denture or implant rehabilitation. This review highlights the impact of oral rehabilitative strategies on brain function and mastication, ultimately informing rehabilitation options that aim to improve masticatory function and, consequently, nutritional status in older adults.

A key theme emerging across these publications is the growing recognition of the close interconnection between oral health and systemic health in the elderly. Evidence increasingly indicates that compromised oral health is not an isolated issue but is linked to chronic conditions prevalent in this age group, including vitamin D deficiency, physical frailty, and cognitive decline. For example, poor oral health can impair chewing and swallowing, resulting in inadequate dietary intake, malnutrition, weight loss, and a diminished quality of life. This interconnectedness highlights the importance of adopting an integrated healthcare approach that combines dental, medical, and nutritional interventions to address the multifaceted needs of older individuals.

Looking ahead, future research should aim to further elucidate these associations, particularly by focusing on the mechanisms underlying the link between oral and systemic health. Developing comprehensive, multidisciplinary care models will be crucial to optimizing health outcomes, preventing nutritional deficiencies, and enhancing quality of life. Such efforts are vital as the global population ages, and the burden of age-related oral and systemic diseases continues to rise.

In summary, these contributions reaffirm that maintaining oral health is a cornerstone of nutritional well-being and systemic health in older adults. They call for heightened awareness, early intervention, and collaborative care strategies to meet the complex needs of this growing demographic. We hope that this Special Issue will inspire continued research and clinical innovation to promote healthy aging and improve the quality of life of older populations worldwide.

